# Meaning and resilience in war-affected populations during crisis

**DOI:** 10.3389/fpsyt.2025.1678205

**Published:** 2025-10-24

**Authors:** Pninit Russo-Netzer, Ricardo Tarrasch, Rotem Saar-Ashkenazy, Jonathan Guez

**Affiliations:** ^1^ C.M.H. Development and Research Institute, Faculty of Psychology, Achva Academic College, Shikmim, Israel; ^2^ School of Education, and Sagol School of Neuroscience, Tel Aviv University, Tel Aviv, Israel; ^3^ Social Work, Ashkelon College, Ashkelon, Israel

**Keywords:** meaning in life, psychological resilience, public mental health, trauma and coping, protective factors, well-being during crisis

## Abstract

**Introduction:**

The unprecedented events of October 7th, 2023, in Israel have profoundly impacted mental health and psychological functioning. This study aimed to explore how protective psychological factors—specifically meaning in life (MIL) and optimism—contribute to resilience in the context of wartime trauma. The study focused on identifying which protective factors most strongly predict resilience and psychological outcomes during collective trauma.

**Methods:**

A mixed-methods design was employed, combining quantitative and qualitative approaches. The quantitative sample included 758 participants from a national cohort. Stepwise multiple regression analyses were conducted to identify significant predictors of resilience from protective factors including social support, prioritizing meaning, presence of meaning, optimism, and psychological distress indicators. In parallel, qualitative data were collected through participants’ written reflections, which were analyzed using thematic analysis to identify sources and perceptions of meaning during the war.

**Results:**

Regression analyses revealed that six factors significantly predicted resilience, explaining 36% of the variance: optimism emerged as the strongest predictor (β = 0.31), followed by psychological distress (β = -0.17), prioritizing meaning (β = 0.13), concern about the state’s future (β = -0.09), presence of meaning (β = 0.10), and social support (β = 0.08). Thematic analysis revealed that participants derived meaning from activities such as volunteering, maintaining daily routines, nurturing relationships, and engaging in spiritual practices, all of which were associated with higher resilience scores.

**Discussion:**

The integration of quantitative and qualitative findings underscores the central role of optimism and meaning-making in psychological resilience during collective trauma. Optimism emerged as the most influential protective factor, while meaning-related orientations and social support provided complementary contributions. The coherence between data sources strengthens construct validity and offers valuable theoretical and practical implications for interventions aiming to foster resilience in contexts of war and crisis.

## 1 Introduction

War leaves lasting scars on individuals and societies alike. On October 7th, 2023, a brutal and unprecedented attack took place in southern Israel. Over 1,300 civilians lost their lives, and another 240 were kidnapped and taken hostage. The “Iron Swords” war that followed posed significant challenges to civilians, causing a complex and profound disruption to both societal and individual well-being. These events left enduring marks on mental health and psychological functioning. Studies have consistently demonstrated the detrimental impact of war on the mental health of survivors, soldiers, and civilians exposed to conflict zones (e.g., 1–3). These studies have showed that war-induced trauma is often followed by elevated rates of depression, anxiety and post-traumatic stress disorder (PTSD), which is characterized by a range of traumatic symptoms, encompassing intrusive memories, flashbacks, hypervigilance, and avoidance (Diagnostic statistical manual of mental disorders, DSM-5; 4; in addition, see 5–7; 8). Additionally, studies demonstrate the association between war exposure and heightened risk for substance abuse, further exacerbating psychological distress and impairing overall well-being (e.g., 9, 10). For example, studies on the Ukrainian crisis have illuminated the acute psychological distress, increased rates of PTSD, and significant disruptions to social and community structures caused by the conflict (11–13). Beyond mental health, the consequences of war extend to physical health challenges due to injuries, disabilities, and limited access to healthcare services (e.g., 13, 14). Socially, war disrupts communities, leads to displacement, loss of livelihoods, and exacerbates poverty, thus straining the fabric of society (15). Moreover, the enduring trauma of war can impede post-conflict recovery and reconciliation efforts, prolonging the distressing effects on individuals and communities long after the conflict have ceased (16).

### 1.1 Resilience during war and crisis

The American Psychological Association defines resilience as “the process and outcome of successfully adapting to difficult or challenging life experiences, especially through mental, emotional, and behavioral flexibility and adjustment to external and internal demands” (17). Research on resilience has shifted from viewing it as a stable trait to understanding it as a dynamic process. This approach focuses on how various resources can mitigate the adverse effects of stressors, thereby fostering positive outcomes (18). Supporting this shift, researchers studying resilience in the face of adversity have proposed a framework that emphasizes the dynamic nature of resilience across different time periods—immediate, short-term, and long-term (19). In this framework, the authors discuss how resilience evolves and manifests at various stages in the face of adversity, thus emphasizing the importance of considering temporal elements such as immediate reactions, short-term adaptation, and long-term adjustment in understanding resilience processes. While resilience is often discussed in long-term terms, recent theoretical developments (e.g., 19) highlight that resilience may also manifest in immediate and evolving responses to trauma. Accordingly, we adopt a dynamic, process-oriented view of resilience that allows for the identification of early protective psychological mechanisms in the context of ongoing collective trauma.

Research on the ongoing Russia-Ukraine conflict has shed light on what contributes to resilience on a national and individual level. Data collected before the 2022 Russian invasion of Ukraine showed a strong sense of Ukrainian identity and belonging, which has been further boosted during the conflict, with increased pride, support for independence, and optimism for the future. National resilience in Ukraine has been found to be high, with interpersonal trust and individual resilience being key factors contributing to this. Language spoken, belief in one’s ability to bounce back from adversity, and age also play roles in shaping national resilience. Despite economic challenges, economic losses during the conflict do not seem to have significantly impacted national resilience, suggesting that Ukrainians are united in their determination to face the hardships of the conflict together (20). Looking at individual resilience of the Ukrainian population, research revealed high levels of personal resilience despite significant exposure to threats and stressors. Social support and a sense of purpose were identified as key protective factors, while fear, lack of resources, and social isolation were associated with increased vulnerability (21).

### 1.2 The role of social support in war

Research on the impact of war and conflict offers insights into the role of social support networks and community resilience in mitigating the negative impacts of war on individuals’ well-being. For instance, a qualitative study conducted in post-war Northern Sri Lanka emphasizes that interstate conflicts tend to weaken social fabric, reducing interpersonal and communal trust, and undermining cooperation for the common good. These findings underscore the importance of considering cultural, gender, and spiritual sensitivity in the healing process, along with encouraging access to natural and ancillary resources (22)​. Similarly, a study following the armed conflict in North Gondar, Ethiopia, emphasized the need for holistic support, proper burial of the dead, and re-initiation of social gatherings to help alleviate the existing problems and create a resilient community (23). The level of perceived social support among Syrian refugees who have experienced war was also found to be a direct predictor of later posttraumatic growth (24).

### 1.3 Meaning in life and optimism as contributors to resilience during war

The impact of war on individuals extends beyond immediate physical harm, profoundly influencing their psychological and existential well-being. One critical aspect affected by war is individuals’ sense of meaning in life (MIL). Overall, the construct of MIL encompasses two distinct dimensions: the search for meaning, which involves the active pursuit of significance, making sense and purpose, and the presence of meaning, which relates to the subjective feeling of experiencing meaning in one’s life (25). Presence of meaning refers to an individual’s perception of the meaningfulness of their life, while the search for meaning reflects the motivation and effort to uncover meaning in life (25). Studies have showed the pivotal role of MIL in preserving resilience during adversities; these studies have linked the presence of meaning to enhanced well-being indicators such as positive emotions, life satisfaction, and happiness (26–29). Search for meaning, on the other hand, presents a complex interplay of positive and negative associations, reflecting its dual nature in human experiences and psychological processes. For example, empirical research has found that searching for meaning is associated with less life satisfaction (e.g., 28) and greater anxiety, depression and rumination (e.g., 26), but also with positive outcomes such as open mindedness, drive and absorption (25, 26). While the constructs of prioritizing meaning, meaning-making, and meaning in life are closely related, they reflect distinct psychological processes. *Prioritizing meaning* refers to an intentional life orientation that gives precedence to meaningful pursuits. *Meaning-making* denotes the active process of reinterpreting experiences, particularly in response to trauma, to restore a sense of coherence. *Meaning in life* reflects the subjective experience of life as purposeful and significant, whether through the presence or active search for meaning. In the context of post-traumatic growth, these elements may interact dynamically to support adaptive coping and resilience.

Given the uncertainties and challenges inherent in a situation of war, it is crucial to explore the potential interplay between the presence and search for meaning. This exploration is essential for gaining a deeper understanding of the role of MIL as a protective factor fostering resilience during adversity. Previous research indicates that war and collective trauma can significantly disrupt individuals’ perception of personal and cultural meaning (30). This disruption not only affects their mental health but also impacts their overall resilience and ability to cope with ongoing challenges. Studies have shown that exposure to war-related stressors can lead to a sense of existential crisis, where individuals struggle to find coherence and purpose in their lives (31). Importantly, researchers have observed a direct relationship between finding MIL and experiencing lower levels of psychological distress following trauma (32). Individuals who can derive meaning from their experiences, despite their challenging nature, often exhibit higher levels of resilience and adaptive coping strategies. They may be better equipped to make sense of their circumstances and maintain a sense of purpose that fuels their resilience. Moreover, actively prioritizing meaning in life has been linked to several positive psychological outcomes beyond resilience. Those who emphasize meaning report higher levels of life satisfaction, increased happiness, more frequent experiences of positive emotions, a greater sense of coherence in their lives, heightened feelings of gratitude, and an overall sense of fulfillment (33). This highlights the multifaceted benefits of integrating meaning into one’s life, especially in challenging environments like war-torn regions. In essence, recognizing the importance of MIL not only serves as a coping mechanism but also contributes significantly to individuals’ overall well-being and resilience levels during and after experiencing the trauma of war.

In addition to the role of meaning in life, optimism stands out as another crucial factor contributing significantly to resilience and overall life satisfaction (34). Extensive research has highlighted optimism as a potent psychological resource that plays a pivotal role in helping individuals navigate and overcome adversity. A 37-year longitudinal study focusing on American prisoners of war held in Vietnam during the 1960s and early 1970s sheds light on the profound impact of optimism on resilience (35). The study revealed that optimism emerged as the strongest variable correlated with resilience among these individuals, underscoring its potential as a protective factor in facing and recovering from traumatic experiences. Further supporting optimism’s protective role, research following the Oslo bombing in 2011 highlighted its beneficial effects in mitigating PTSD symptoms (36). Optimism acted as a barrier against the development of certain PTSD symptoms and showed that individuals with a more optimistic disposition exhibited lower initial levels of PTSD symptoms, suggesting that optimism not only promotes resilience but also helps buffer against specific psychological challenges associated with trauma exposure. These findings underscore optimism’s multifaceted impact on mental health outcomes, resilience, and adaptive coping strategies. Optimistic individuals tend to maintain a positive outlook, maintain hope for the future, and actively seek solutions to problems they encounter. These adaptive cognitive and emotional processes significantly contribute to their ability to bounce back from setbacks, maintain psychological well-being, and sustain overall life satisfaction even in the face of adversity (e.g., 37, 38). In addition to examining protective resources, we assessed key indicators of psychological distress commonly observed in war-affected populations—including depression, anxiety, stress, and loneliness—to capture a fuller picture of the psychological impact of collective trauma and the potential buffering role of meaning and optimism.

Taken together, the literature suggests that protective psychological factors—such as meaning in life and optimism—can buffer the negative impact of war-related stressors on mental health. Prioritizing meaning and receiving social support may enhance these protective factors, thereby promoting resilience and reducing distress. However, the specific mechanisms through which these factors interact during times of collective trauma, particularly in the context of acute and ongoing war, remain underexplored. Building on this foundation, the current study aims to examine whether the presence of meaning and optimism mediate the associations between prioritizing meaning and social support and psychological outcomes, including resilience and distress. We also explored individuals’ subjective experiences to gain a deeper understanding of meaning-making during wartime. Accordingly, the study aims to advance our understanding of resilience and meaning-making processes in the face of collective trauma through an integrative model grounded in meaning orientation, utilizing a mixed-methods approach. Its uniqueness lies in the real-time examination of psychological experiences within Israel’s diverse society during the acute phase of national trauma.

### 1.4 The present study

The conflict between Hamas and Israel, notably intensified by the attack on October 7th, presents a harsh reality that has significantly impacted the well-being and resilience levels of both Israelis and Palestinians. The conflict has spawned a pervasive cycle of violence, elevating levels of stress, trauma, and daily life adversity amidst an atmosphere of war. The circumstances have been particularly traumatic due to the substantial civilian casualties and the extreme brutality inflicted upon the victims, including vulnerable populations such as children and the elderly. The displacement, ongoing uncertainty, and close proximity to violence are conducive to a collective psychological trauma (39). These events resulted in profound and widespread psychological distress. Despite the growing interest in resilience, research examining real-time protective mechanisms, such as meaning prioritization, MIL, and optimism, in the context of collective trauma, remains scarce. Our study addresses this important gap. The current study, thus, seeks to explore the construct of resilience within this war-torn context, examining how protective factors such as MIL and optimism may promote resilience in the context of war. It is based on the assumption that the destabilization wrought by war activates various protective psychological mechanisms, with some individuals drawing on optimistic thinking, meaning-making processes, and social support more effectively than others, leading to differential levels of resilience and psychological functioning. Grounded in theoretical frameworks of meaning-oriented coping and resilience in contexts of ongoing collective trauma, our central hypothesis was that resilience during wartime would be significantly predicted by several protective psychological factors, including optimism (the ability to maintain hopeful and goal-directed thinking), presence of meaning (a sense of coherence and purpose), prioritizing meaning (intentional orientation toward meaningful activities), and social support, while being negatively predicted by psychological distress and concern about the future.

To complement the quantitative analysis and deepen our understanding of how individuals experience and respond to a national emergency, we also incorporated an exploratory qualitative component involving two open-ended questions: (1) What activities provided participants with a sense of meaning during the war? and (2) What meaning did they ascribe to the events of October 7th?

## 2 Method

### 2.1 Participants and procedure

Recruitment of participants started 5 days after the events of October ^7th^ in Israel (on 12/10/2023), and ended on 09/11/2023. Data were collected through snowball sampling from Israeli citizens above the age of 18 by means of a computerized survey distributed via social media. Participants answered to socio-demographic and background questions, followed by the study items presented below. In total, 758 participants (351 men, 403 women and 4 other) provided full surveys and were included in the analysis. The age of the participants ranged between 18 and 79 with a mean age of 39.78 (s.d. 13.65). 26.0% of the participants were single, 67.6% married/in relationship, 5.7% divorced and 0.7% widow. The economic status of the participants was 44.8% below average, 25.1% average and 30.1% above average. The educational status of the participants was 0.5% without education, 1.3% elementary school, 17.4% middle/secondary school, 72.3% academic, 8.5% professional. The religiosity level of the participants was 56.1% secular, 22.4% traditional, 15.4% religious and 6.1% ultra-orthodox. All participants provided written informed consent for participating in the study. The study was reviewed and approved by the institutional review board of Achva Academic College prior to data collection [No. 0175]. At the time of data collection, all participants were residing in Israel during the early phase of the war and were therefore affected, to varying degrees, by the collective trauma context.

### 2.2 Instruments

#### 2.2.1 Social support

The 12-items Multidimensional Scale of Perceived Social Support (MSPSS, 40) was used to measure perceived Social Support. The MSPSS is composed of three subscales, each addressing a different source of support: Family (e.g., “My family really tries to help me”, friends (e.g., “I can talk about my problems with my friends”), and significant other (e.g., “There is a special person in my life who cares about my feelings”) on a scale ranging between 1 (“very strongly disagree”) to 7 (“very strongly agree”). Sub-scales scores are calculated by calculating the mean value for each sub-scale and the total scores is composed from the mean value of all 12-items, with higher scores indicating higher levels of perceived social support. Cronbach’s alpha values for the MSPSS scores were reported as.91 (significant other),.87 (family),.85 (friends) and.88 for the total MSPSS score (40). Cronbach’s alpha for total MSPSS scores in the current study was.94 (.91,.94,.92 for the family, friends and significant other sub-scales, respectively).

#### 2.2.2 Prioritizing meaning

The prioritizing meaning scale is composed of 12 items aimed to measure the extent to which individuals intentionally act and organize and accordingly make decisions in order to experience more meaning in their life (33). Participants are requested to respond to each item (e.g., “I prefer to engage in activities which are related to the sense of meaning in my life”) on a 9-point scale (1=disagree strongly, 9=agree strongly). A total score is obtained by calculating the mean score from all items. Cronbach’s alpha was reported to be α =.95 (33). In the current study alpha was.95.

#### 2.2.3 Meaning in life

The Meaning in Life Questionnaire (MLQ; 25) was used to measure the “search for” (e.g., “I am always looking to find my life’s purpose”) and “presence of” (e.g., “I understand my life’s meaning”) meaning in life. Each subscale is comprised of five items, with answers to both subscales raging between 1 (“absolutely untrue”) and 7 (“absolutely true”). Total scores were obtained according to the questionnaire guidelines and range between 5 and 35. Cronbach’s alpha for presence and search scales were reported as ~.85 (for both scales, 25). Cronbach’s alpha in the current study was.84 for “search” and.9 for “presence”.

#### 2.2.4 Optimism

The Life Orientation Test (LOT, 41) was used to measure optimism. Participants were asked to respond to each item (e.g., “In uncertain times, I usually expect the best”) on a 5-point Likert scale, ranging between 0 (“strongly disagree”) to 4 (“strongly agree”). Following recoding of reversed items and omission of filler items, a total score is obtained by summing all designated 8 items; Higher scores represent higher level of optimism. Cronbach’s alpha for 8-items LOT was reported as.76 (41). Cronbach’s alpha in the current study was.84.

#### 2.2.5 Resilience

The Connor-Davidson Resilience Scale (CD-RISC; 42) was used in order to measure resilience. In the current study we employed the 10-items validated version (43) which is a self-report scale intended to measure resilience using 10-items (e.g. “I can deal with whatever comes”) on a Likert scale ranged between 0 (“not true at all”) to 4 (“true nearly all the time”). Total scores are calculated by summing all items with higher scores indicating higher resilience. Cronbach’s alpha for the 10-items CD-RISC was reported as.85 (43). Cronbach’s alpha in the current study was.9.

#### 2.2.6 Loneliness

Loneliness was measured via a 3-items scale (44). Participants were asked to reply to three questions; how often do you feel that you lack companionship, how often do you feel left out and how often do you feel isolated from others. For each item, participants were asked to respond on a 1 (“hardly ever”) to 3 (“often”) scale, and the total score was calculated by summing all three items. Cronbach’s alpha for 3-items loneliness scale was reported as.72 (44). Cronbach’s alpha in the current study was.91.

#### 2.2.7 DASS

The 21-items Depression Anxiety Stress Scales (DASS-21, 45, 46) was used to measure the negative emotional states of depression (e.g., “I couldn’t seem to experience any positive feeling at all”), anxiety (e.g., “I felt scared without any good reason”) and stress (e.g., “I tended to over-react to situations”). Each of the three DASS-21 sub-scales contains 7 items; All ranging between 0 (“never”) to 3 (“almost always”). Calculation is performed by summing all 7 items belonging to each sub-scale, and all 21 items for the total score. The DASS-21 is based on a dimensional conception of psychopathology, and scores emphasis the degree to which participants experience symptoms in each sub-scale. Cronbach’s alpha for the-21 DASS scales were reported as.93 (total scale), and.88,.82,.90 (for the depression, anxiety and stress scales, respectively; 45). Cronbach’s alpha in the current study was.94 (.86,.88 and.9 for depression, anxiety and stress sub-scales, respectively).

All of these measures have been previously validated.

#### 2.2.8 Concern

The nature of October 7^th^ events and the war that followed them act as a collective trauma. Thus, a single question, especially written for the purposes of this study was used. Participants were asked “What is the extent of your concern about the future of the State of Israel in light of the ‘Iron Swords’ war?, using a scale ranging between 1 (Not worried) and 7 (Extremely worried).

#### 2.2.9 Open-ended questions

Participants were also asked to response to open-ended questions regarding (a) their experiences related to which activities give them a sense of meaning since the October 7^th^ events and during the war, and (b) and whether they feel that there is any meaning to the October 7^th^ events and the following war, and if so, to describe which meaning they ascribe to them.

### 2.3 Quantitative data analysis

To address the study’s two central aims—(1) identifying which protective factors significantly predict resilience and other psychological outcomes, and (2) exploring participants’ sources and perceptions of meaning during wartime—we employed a mixed-methods approach. The quantitative component utilized stepwise multiple regression analysis to identify the most significant predictors of resilience from our theoretical model.—we employed a mixed-methods approach. The quantitative component utilized a regression analysis. In parallel, the qualitative component explored participants’ subjective narratives, and was further connected to the quantitative findings through comparative analyses (e.g., chi-square, t-tests). Each analytic strategy was selected to align with the corresponding research question and contribute to a coherent understanding of psychological resilience in the context of collective trauma.

A stepwise multiple regression analysis was conducted to examine the extent to which social support, prioritizing meaning, presence of meaning in life, search for meaning in life, optimism, loneliness, psychological distress, and concern about the future of the state predicted resilience. Variables were entered in a stepwise manner, allowing only predictors that contributed significantly to the model to be retained.

To ensure that the sample size was sufficient for this analysis, we conducted an *a priori* power analysis for a multiple regression model with up to eight predictors. Following Cohen’s (47) conventions for effect size in multiple regression, we used *f* ^2^ (where *f* ^2^ = *R* ^2^/(1− *R* ^2^)). An a-priori power analysis for a fixed-model regression (overall *R* ^2^ different from 0), with α = .01, power (1–β) = .80, and a small effect of *f* ^2^ = .02 indicates a required sample of ≈ 490 cases when testing ~8 predictors. Our final sample of 760 thus exceeded the requirement by a wide margin.

### 2.4 Qualitative data analysis

The two open-ended questions underwent thematic analysis grounded in a social constructionist framework (48). Following Braun and Clarke’s (49) six-phase approach, the analysis began with a thorough familiarization process, in which two of the authors independently read and re-read the responses to gain a deep understanding of their content. Initial codes were then generated inductively by each coder, identifying recurring patterns and meaningful units in the text. Each sentence could be coded under multiple categories, reflecting the richness and multidimensionality of participants’ experiences.

Next, the coders met to compare and discuss their codes, and through an iterative process of dialogue and refinement, organized the codes into coherent candidate themes. These themes were then reviewed in relation to the full dataset, ensuring internal consistency and distinctiveness. Following this, the themes were clearly defined and named, and representative quotations were selected to illustrate each theme. Final coding was conducted based on the agreed thematic framework.

To ensure reliability, inter-rater agreement was calculated using Cohen’s kappa (50), separately for each theme. The question regarding activities that provided participants with a sense of meaning during the war yielded 11 themes, with kappa values ranging from.61 to.96 (seven themes above.80). The question regarding the meaning of the October 7th events yielded 8 themes, with kappa values ranging from.68 to.92 (four themes above.80). A summary of the identified themes and examples of coded responses appear in **Table 1**. Examples to each of the category are presented in the Results section.

**Table 1:** Percentages of appearance of themes related to activities that granted participants a sense of meaning during the war.

Theme	% appearance (both agree)
Contribution and volunteering	41.3%
Relationships	36.2%
Daily routine	17.4%
Physical activity	14.7%
Hobbies and leisure	12.2%
Religious/spiritual practices	7.6%
Self-control and relaxation	5.2%
Learning and reflection	5.1%
No meaning/nothing	2.9%
Social networking and explanation	2.7%
Nature	1.1%

Percentages of Appearance of the Themes Related to Activities which Grant the participants a sense of meaning during the war. Percentages sum more than 100%, as each answer could be categorized into more than one theme.

Furthermore, gender, religiosity levels and being personally affected by war (yes\no) differences between the themes that emerged from the two open-ended questions were assessed using chi-square for independence tests. For each of the themes of the two open-ended questions, T-tests for independent samples were calculated where the independent variable was the appearance of the theme (yes versus no) and the dependent variables were the quantitative measures collected in the study. Due to the number of tests assessed and taking into consideration the exploratory nature of some of the analyses, alpha was set at.01.

## 3 Results

### 3.1 Quantitative results

**Table 2** presents the means and standard deviations of the dependent variables on the study, available for all 758 participants.

**Table 2:** Study variables means and standard deviations.

Variable	Mean	Std. Dev.
MSPSS-Sig other	5.76	1.37
MSPSS-Family	5.41	1.44
MSPSS-Friends	5.28	1.52
Prioritizing meaning	6.55	1.52
MIL presence	21.34	7.32
MIL-search	23.82	6.67
Optimism	20.35	5.62
Resilience	35.48	7.07
Loneliness	7.64	3.18
DASS	42.21	13.78
Concern	5.01	1.74

**Table 3** displays Pearson correlations among study variables.

**Table 3:** Pearson correlations between study variables.

Variable	1	2	3	4	5	6	7	8	9	10	Gender^1^	Age	Marital^2^	Relig	Economic	Education
1-MSPSS-Sig other											.21**	-.01	.32**	-.06	.01	.04
2-MSPSS-Family	.69**										.08*	.06	.28**	-.02	.09*	.04
3-MSPSS-Friends	.67**	.57**									.15**	-.05	.12**	-.08*	.03	.03
4-Pioritizing MIL	.36**	.34**	.36**								.13**	.08	.10*	.13**	.03	.11*
5-MIL presence	.31**	.32**	.29**	.66**							.09	.07	.16**	.25**	.02	.04
6-MIL-search	.06	.07	.11	.45**	.46**						.07	-.08	-.07	.17**	-.11*	-.07
7-Optimism	.40**	.37**	.36**	.48**	.53**	.09					.09	.09*	.16**	.13**	.14**	.14**
8-Resilience	.27**	.24**	.36**	.39**	.42**	.12**	.53**				-.08	.06	.08	-.03	.18**	.06
9-Loneliness	-.46**	-.44**	-.53**	-.27**	-.27**	.07	-.40**	-.31**			-.07	-.12*	-.26**	.07	-.09	-.08
10-DASS	-.16**	-.24**	-.19**	-.13**	-.21**	.08	-.38**	-.36**	.33**		.19**	-.24**	-.09	-.06	-.20**	-.08
11-Concern	.04	.02	-.02	.02	-.06	.06	-.16**	-.18**	.09	.26**	.19**	-.01	.04	-.17**	-.07	.04

1 – only men and women were used, the correlation index being point-biserial.

2 – only single and married participants were used, the correlation index being point-biserial.

* p <.01.

**p <.001.

The final regression model was significant, *F*(6, 753) = 68.57, *p* <.001, and explained 36% of the variance in resilience (*R*² = .36, adjusted *R*² = .35). Six predictors were retained: optimism, psychological distress, prioritizing meaning, concern, meaning in life presence, and social support. **Table 4** presents the standardized regression coefficients (*β*) of the significant predictors.

**Table 4:** Standardized regression coefficients (β) for predictors of resilience.

Predictor	*β*	*t*	*p*	*ΔR² (%)*
Optimism	.31	7.85	<.001	28.1
Psychological distress	–.17	-5.06	<.001	2.9
Prioritizing meaning	.13	3.04	.002	2.8
Concern about the future	–.09	-2.73	.006	0.6
Meaning in life (presence)	.10	2.43	.015	0.5
Social support	.08	2.39	.017	0.5

The overall regression model was significant, *F*(6, 753) = 68.57, *p* <.001, explaining 36% of the variance in resilience (*R*² = .36, adjusted *R*² = .35).

### 3.2 Qualitative results

To complement the quantitative findings, the qualitative strand examined participants’ responses to two open-ended questions—one regarding the kinds of activities that provided them with a sense of meaning during the war, and another about the meaning they ascribed to the October 7th events. Together, these insights provide a broader picture of how individuals navigated meaning-making in the context of collective trauma.

For the question about whether they feel that there is any meaning to the October 7^th^ events, the categories are presented in **Table 5**.

**Table 5:** Percentages of appearance of themes related to the meaning of the October 7^th^ events for participants. .

Theme	% appearance (both agree)
Security meaning	21.2%
National unity	19.8%
No meaning/nothing	16.6%
Political meaning	16.4%
Existential meaning	15.4%
Awakening, awareness, perception changes	12.5%
Personal and spiritual meaning	6.1%
Encounter with evil	1.5%

Here are some examples of participants’ sentences categorized under each theme:

*No meaning/nothing*: “It’s hard to find anything that gives a sense of meaning during this difficult time”, “I have no meaning to life!!”; *Relationships*: “To be with my family and friends and get support”, “Being close to family, especially my children, gives me strength”, “supporting people and friends is a priority for me now”; *Religious/spiritual practices:* “Praying”, “Meditation”, “Studying holy scriptures”, Engaging in prayer with greater enthusiasm”; *Contribution and volunteering:* “Volunteering in hospitals to support the wounded”, “Helping displaced civilians and children”, “Contributing to people who need comfort or encouragement”, “Helping people around me who are suffering from the situation”; *Physical activity*: “Sports”, “Exercise and yoga to calm the mind”; *Hobbies and leisure:* “Knitting and reading”, “Playing the guitar”; *Daily routine*: “Working and making meals”, “Training at the gym, walking to work every day, walks on the beach or in the park”.; Social networking and explanation: “Translating survivors’ stories to different languages to post in social media to increase awareness to the horrible events”, *Learning and reflection:* “Studying and listening to inspiring lectures”, “Journaling about my emotions”, “Reflecting about what matters most in life in face of this crisis”; *Nature:* “Going outside to see the sunlight”, “Being in nature”; *Self-control and relaxation*: “Breathing exercises”, “Calming myself”.

Here are some examples of participants’ sentences categorized under each theme: *No meaning/nothing:* It’s hard to believe that there is meaning for what happened”; *Security meaning:* “It has proven that we are not safe in our own home in our own country”; “It’s time to wake up and open our eyes—if we don’t know how to be strong, unfortunately we’ll be in such situations again”, “Shows the need for Israel to have a strong army*”; Existential meaning:* “Once again we need to fight for our existence”; *Awakening, awareness, perception changes:* “It’s a wake up call to change perspectives”, “A trigger for reconsideration, redirection”, “Paradigmatic change in who is a partner, enemy, or murderer”; *National unity:* “It has proven that we need to reunite as a nation beyond internal differences or disputes”, “Unity is needed”, “The people’s unity finally renewed”; *Personal and* sp*iritual meaning:* “I feel that there must be a spiritual meaning to what happened, but don’t see the greater picture yet”, “It has reminded me the things that are truly important in life; *Political meaning*: “It has proven that the current government needs to be changed. I lost trust”, “The current government dealt with nonsense and neglected security. What happened is the punishment for arrogance and complacency”; *Encounter with evil:* “It made me feel shaken and horrified to feel such pure evil that seeks to destroy. We will never be the same”. “It reminded me that human evil still exists, and it’s so sad”.

**Table 1** presents the percentage of participants among which both raters agreed on the theme appearance, regarding the open-ended question of activities which grant the participants a sense of meaning during the war.

**Table 5** presents the percentage of participants among which both raters agreed on the theme appearance regarding the open-ended question related to the meaning of the October 7^th^ events for the participants.

**Figure 1:**
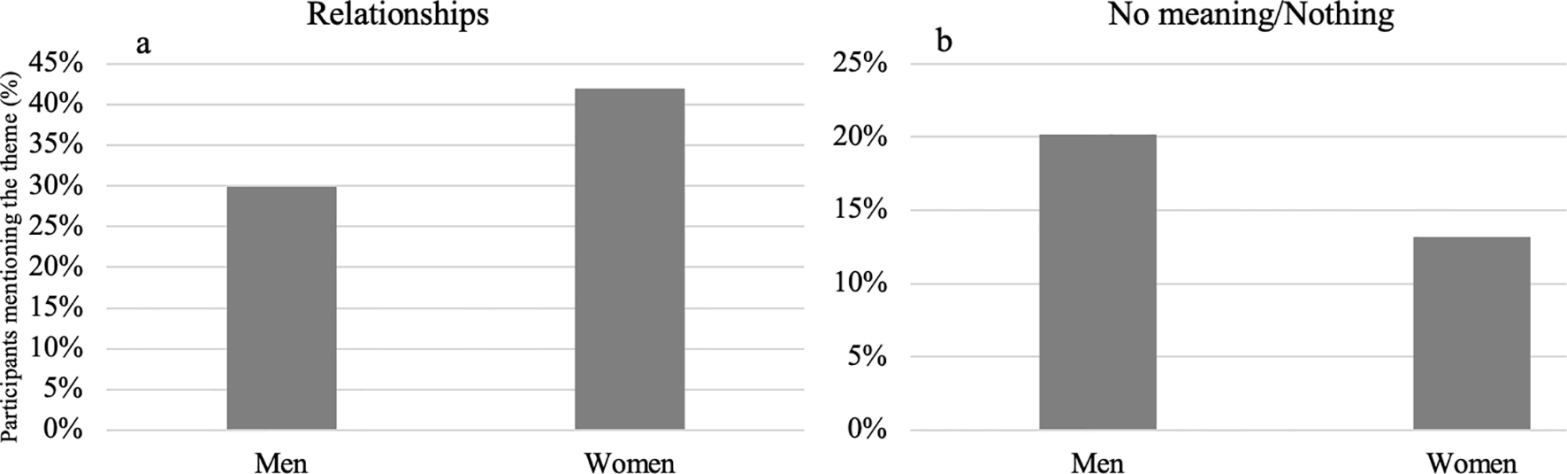
Gender significant differences between the themes that emerged from the open-ended question of activities which grant participants a sense of meaning during the war: **(a)** Relationships; **(b)** no meaning/nothing.

Some gender differences were obtained for the themes that emerged from the open-ended questions. As can be seen in **Figure 1A**, women significantly mentioned more relationships ( $\chi_{\left(1\right)}^{2}$ = 11.52, *p* <.001) among activities which grant the participants a sense of meaning during the war, while men (**Figure 1B**) significantly provided more answers related to no meaning ( $\chi_{\left(1\right)}^{2}$ = 6.84, *p* = .009) in the question about the meaning of the October 7^th^ events.

**Figure 2:**
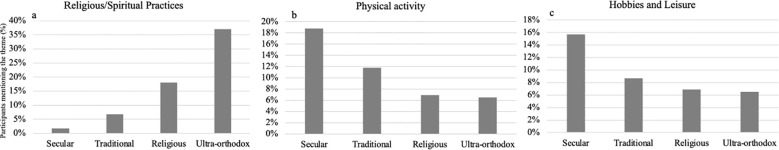
Religiosity significant differences for themes that emerged in the open-ended question of activities which grant participants a sense of meaning during the war: **(a)** Religious/spiritual practices; **(b)** physical activity; **(c)** hobbies and leisure.

Religiosity differences were observed for the themes that emerged in the open-ended question of activities which grant the participants a sense of meaning during the war, significant differences appeared between religiosity groups for religious/spiritual practices ( $\chi_{\left(3\right)}^{2}$ = 96.79, *p* <.001, **Figure 2A**), physical activity ( $\chi_{\left(3\right)}^{2}$ = 14.75, *p* = .002, **Figure 2B**), and hobbies and leisure ( $\chi_{\left(3\right)}^{2}$ = 11.00, *p* = .012, **Figure 2C**).

**Figure 3:**
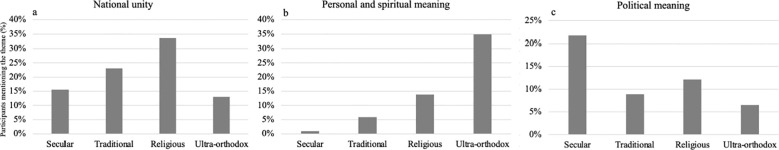
Religiosity significant differences in themes that emerged from the open-ended question regarding the meaning of the October 7^th^ events for participants: **(a)** National unity; **(b)** personal and spiritual meaning; **(c)** political meaning.

Within the themes that emerged from the open-ended question regarding the meaning of the October 7^th^ events, significant differences appeared between religiosity groups for national unity ( $\chi_{\left(3\right)}^{2}$ = 21.03, *p* <.001, **Figure 3A**), personal and spiritual meaning ( $\chi_{\left(3\right)}^{2}$ = 97.67, *p* <.001, **Figure 3B**), and political meaning ( $\chi_{\left(3\right)}^{2}$ = 20.62, *p* <.001, **Figure 3C**).

T-tests for independent samples with 756 degrees of freedom, revealed significant differences between the appearance of themes (yes versus no) related to activities which grant the participants a sense of meaning during the war, and quantitative variables. In total 30 out of 121 comparisons (24.8%) were significant (*p* <.01) which is markedly larger than the number of comparisons expected to be significant by chance (1-2). Effect size analyses further revealed that 22 of the significant effects were in the small-to-moderate range (Cohen’s d = 0.2–0.5), 2 were in the moderate-to-large range (d > 0.5 and up to 0.8), and 6 reflected large effects (d > 0.8). Cohen’s d of significant differences are presented in **Table 6**. Bold numbers represent cases where the appearance of the theme was accompanied by lower values in the dependent variable (e.g. participants with the theme “No meaning/nothing” had significantly lower social support from significant others - MSPSS-Sig other, as compared with participants without that theme). Non-bold numbers represent cases where the appearance of the theme was accompanied by higher values in the dependent variable (e.g. participants with the theme “Relationships” had significantly higher social support from significant others - MSPSS-Sig other).

**Table 6:** Cohen’s d for significant differences in dependent variables between participants who did and did not relate to each of the themes of activities that granted a sense of meaning during the war.

	MSPSS-Sig other	MSPSS-Family	MSPSS-Friends	Prioritizing MIL	MIL presence	MIL-search	Optimism	Resilience	Loneliness	DASS	Concern
No meaning/nothing	**0.64**			**1.19**	**1.26**	**0.81**	**1.07**				
Relationships	0.38	0.33	0.27	0.20	0.21		0.30				
Religious/spiritual practices				0.39	0.70	0.49					**0.39**
Contribution and volunteering	0.33	0.21	0.34	0.50	0.35		0.29	0.37	**0.30**	**0.22**	
Physical activity											
Hobbies and leisure											
Daily routine											
Social networking and explanation											
Learning and reflection											
Nature			1.17						**0.95**		
Self-control and relaxation	0.49	0.42		0.36			0.43				

T-tests for independent samples with 756 degrees of freedom, revealed significant differences between the appearance of themes (yes versus no) related to the meaning of the October 7^th^ events for participants, and quantitative dependent variables. Nineteen out of 88 of comparisons (21.6%) were significant (*p* <.01) which is markedly larger than the number of comparisons expected to be significant by chance (about 1). Among these effects, 13 were in the small range and 6 in the medium range (Cohen’s d). Cohen’s d of significant differences are presented in **Table 7**. Bold numbers represent cases where the appearance of the theme was accompanied by lower values in the dependent variable, while non-bold numbers represent cases where the appearance of the theme was accompanied by higher values in the dependent variable.

**Table 7:** Cohen’s d for significant differences in dependent variables between participants who did and did not relate to each of the themes of meaning of the October 7^th^ events.

	MSPSS-Sig other	MSPSS-Family	MSPSS-Friends	Prioritizing MIL	MIL presence	MIL-search	Optimism	Resilience	Loneliness	DASS	Concern
No meaning/nothing	**0.30**	**0.30**		**0.53**	**0.46**		**0.53**	**0.43**	0.27		
Security meaning											
Existential meaning											0.50
Awakening, awareness, perception changes				0.30							
National unity	0.27		0.27	0.54	0.53	0.43	0.48	0.44			
Personal and spiritual meaning					0.57		0.51				
Political meaning				0.29							
Encounter with evil											

## 4 Discussion

The current study explored the association between protective factors such as MIL, social support and optimism to resilience during war and adversity. More specifically, we hypothesized that resilience and overall psychological symptomology can be predicted by social support, prioritizing meaning, MIL, and optimism. Anchored in process-oriented framework of resilience and meaning-making during trauma (e.g., 19, 33, 51), the regression analyses delineate a coherent constellation of protective and risk correlates of resilience during an ongoing collective trauma. *Prioritizing meaning* was conceptualized as a motivational orientation that precedes and activates internal resources such as *optimism* and *presence of meaning*, rather than resulting from them. Empirical studies show that intentionally organizing one’s life around meaning can enhance both optimism and the experience of meaning (33, 52). The findings suggest that above all, optimism functions as a central resource that orients individuals toward future-directed engagement, while lower psychological distress, meaning-related orientations (prioritizing meaning and the felt presence of meaning), and perceived social support each contribute unique, complementary associations with resilience. Concern about the state’s future shows a modest negative link with resilience, underscoring the erosive impact of collective uncertainty on psychological resources. Taken together, these patterns portray resilience as a multidimensional configuration—cognitive–emotional steadiness, meaning orientations, and social connectedness—rather than the effect of a single pathway.

These findings shed light on the importance of actively coping with adverse situations such as war, through the proactive organization of daily routines to include meaningful activities (i.e., prioritizing meaning) and seeking social support. In the present context, optimism likely indexes a trait–state composite: a relatively stable dispositional expectancy together with a context-sensitive, appraisal-like component that may be temporarily amplified by mobilized social support and meaning-oriented engagement. Future work should adopt designs that separate state-like variation from trait-like stability (e.g., longitudinal cross-lagged or latent-change models; ecological momentary assessment). In parallel, micro-interventions that temporarily bolster optimistic expectancies and alternative specifications (e.g., modeling optimism as a moderator) can provide convergent evidence. Such designs would more decisively test whether, when, and for whom optimism functions primarily as a proximal appraisal process, a trait-like resource, or their interaction.

Previous studies indicated that intentionally looking for situations, activities and relationships that can lead to naturally-occurring sense of meaning through prioritizing meaning which contributes to individuals’ well-being (33, 52–54). In the context of the present study, it may be suggested that adopting an optimistic outlook on life may contribute to adaptive coping and buffer against the negative psychological impacts of war-related stressors and trauma. Although optimism is traditionally conceptualized as a relatively stable dispositional trait (41), recent research suggests that it may also include state-like aspects that are sensitive to situational influences, particularly during crises (37, 55). In the current study, we propose that perceived social support and the prioritization of meaning serve as activating conditions that momentarily amplify optimistic thinking. These influences are not assumed to change trait optimism, but rather to facilitate adaptive, future-oriented cognitions in response to acute stress. This interpretation aligns with contemporary resilience frameworks that view adaptation as the result of an interaction between enduring dispositions and flexible, context-dependent processes.

The ability to find meaning or make sense of traumatic experiences was found to be associated with better psychological adjustment and lower levels of depression/anxiety/stress. Deriving meaning from traumatic experiences can foster a sense of self-worth, mastery, and control; these in turn, contribute to resilience and better psychological outcomes (56). Furthermore, optimism appears to play a protective role against the adverse impacts of life’s challenges. Numerous studies have highlighted the positive influence of dispositional optimism in mitigating the detrimental effects of different stressors on overall well-being (for example, see 57–60).

The qualitative analysis provided valuable insights into the activities and experiences that helped participants find meaning and purpose during the challenging circumstances of war. More specifically, the findings suggested that the most prevalent themes related to activities which grant the participants a sense of meaning during the war were contribution and volunteering, relationships, daily routine, physical activity and religious/spiritual practices. The dominance of engaging in activities that contribute to the well-being of others or the community such as contribution and volunteering may foster a sense of purpose and meaning, provide them a sense of agency, impact, and personal significance, amidst the chaos, adversity and uncertainty of war, which can be psychologically beneficial. Engaging in prosocial behaviors has been found to make a significant contribution to meaning in life (e.g., 61), and to life satisfaction that seems to be enduring and less susceptible to adaptation (62). Relationships and social connections may serve to provide a sense of belonging, emotional support, and shared experiences (63), which can be highly meaningful during wartime. Relationships and social connections may also offer a sense of meaning and purpose through caregiving, emotional support, and shared goals or values (e.g., 64). Maintaining a daily routine can provide a sense of normalcy, structure, order, and continuity, even in the midst of disruption caused by war. Routine activities can serve as a source of comfort, familiarity, and control, which can contribute to a sense of meaning and purpose (e.g., 65, 66). Additionally, engaging in physical activities or exercise can be a means of self-care, stress relief, and maintaining a sense of control over one’s physical and mental well-being (e.g., 67), particularly during wartime. For some individuals, religious or spiritual practices can serve as a source of meaning, comfort, and guidance during challenging times like war. These practices can provide a sense of connection to a higher power, a belief system, or a community of faith, which can offer hope and a sense of purpose, and maintain resilience in the face of adversity (e.g., 68).

Exploring specific relationships, *Contribution and volunteering* was concomitant with higher social support of all sources, MIL prioritizing and presence, optimism, and resilience and lower loneliness and depression/anxiety/stress. Participants mentioning *Relationships* showed higher social support of all sources, MIL prioritizing and presence and optimism. Interestingly those who mentioned Self-control and relaxation reported higher social support from those in closer circles: significant other and family but not from friends, together with higher Prioritizing MIL and Optimism. Participants who related to *Religious/spiritual practices* reported more MIL prioritizing, presence but also search, together with less concern. The theme of *Nature* was related to higher social support only from friends and less loneliness. Finally, participants who mentioned that nothing grant them a sense of meaning during the war felt less social support from significant others, presented less MIL prioritizing, presence and search and were less optimistic. Overall, this suggests that engaging in activities that contribute to others, maintaining relationships, adhering to routines, engaging in physical activities, and drawing from religious or spiritual practices can all provide a sense of meaning and purpose, which can be highly beneficial for psychological well-being and resilience.

Gender differences were also observed. Women significantly mentioned more relationships among activities that provide a sense of meaning during the war, while men gave more responses indicating a lack of meaning in their answers regarding the significance of the October 7th events. This is consistent with previous studies indicating that men are more inclined towards coherence and making sense, while women were higher for mattering, interpersonal relationships and connection (69–71). Future studies may further explore the potential underlying mechanisms of such gender differences and the similar and distinct ways in which individuals cope with adverse and challenging times such as war.

Differences in levels of religiosity were also found. Individuals with higher levels of religiosity, such as the ultra-orthodox and religious participants, reported engaging in more religious and spiritual practices as activities that grant them a sense of meaning during war. It may be suggested that their faith and religious beliefs serve as primary sources of meaning and purpose, offering comfort and guidance in challenging times (68, 72, 73). Secular and traditional individuals were more likely to mention physical activities as sources of meaning during war, which appear to provide them with a sense of achievement, personal growth, and self-care, that may benefit their overall well-being and sense of purpose (74, 75). Moreover, secular individuals also tended to emphasize hobbies, leisure activities, and personal interests as meaningful pursuits during war. These activities may offer self-expression, and personal fulfillment, serving as effective coping mechanisms and distractions from the stresses of war (76, 77). Further exploration of this direction may enable to shed light on diverse coping strategies and sources of resilience among individuals facing challenging circumstances such as war.

When exploring the meaning attributed to the October 7th events by participants, distinct patterns emerged based on their religious beliefs and social support networks. Religious and traditional participants tended to associate the events’ meaning with the importance of national unity, highlighting a more collective interpretation. In contrast, ultra-orthodox participants emphasized more personal and spiritual meanings, reflecting a deeper connection to their faith and individual beliefs. Secular participants, on the other hand, leaned towards a more concrete-political interpretation of the events. Interestingly, participants who felt that the events held no meaning reported lower levels of social support from significant others and family members. They also expressed reduced prioritization of MIL, lower MIL presence, diminished optimism, fewer close relationships, and increased feelings of loneliness. The theme of national unity was prevalent among participants who reported higher levels of social support from significant others and friends, prioritized MIL, and had a strong sense of MIL presence and search, optimism, and resilience. This suggests that a sense of connectedness and support from others may influence how individuals perceive and derive meaning from collective events. Conversely, personal and spiritual meanings were associated with higher levels of MIL presence and optimism, reflecting the importance of individual beliefs and spiritual perspectives in finding personal meaning and maintaining a positive outlook. One possible explanation for these findings is that individuals draw meaning from events based on their cognitive and emotional resources, which are influenced by their social support networks, beliefs, and coping strategies. Those with strong social connections and a sense of purpose may interpret events in a more positive and meaningful light, whereas those lacking social support may struggle to find significance or derive meaning. These insights highlight the interconnectedness between social support, belief systems, and the perception of meaning in the face of challenging events.

The present study offers a multidimensional contribution to the understanding of resilience in war-affected populations by illuminating the dynamic interplay between internal meaning-making processes, social support, and optimism during an ongoing collective trauma. Rather than approaching these constructs in isolation, our findings suggest that resilience emerges through the interaction of external resources—such as perceived social support—and internal orientations—such as the intentional prioritization of meaning and dispositional optimism. Optimism, in particular, stood out as a central psychological pathway linking both meaning and social connectedness to greater resilience and reduced psychological distress. While the presence of meaning played a more modest mediating role in the quantitative models, its prominence in participants’ narratives points to the evolving and context-sensitive nature of meaning-making in times of acute crisis. This supports and extends existing theoretical models by positioning resilience as a flexible, intentional process shaped by both inner and relational resources.

By integrating quantitative and qualitative findings, this study provides a richer, more contextualized view of how individuals navigate adversity. The convergence between self-reported data and narrative accounts reinforces the central role of meaning-making, optimism, and connection in coping with trauma. Participants described personally meaningful activities—such as volunteering, spiritual practice, maintaining daily routines, and nurturing relationships—as sustaining forces during the war. These insights not only affirm the construct validity of the studied variables but also highlight how meaning is embodied in everyday practices. At the same time, points of divergence—such as the discrepancy between the statistical weight of presence of meaning and its narrative salience—demonstrate the added value of mixed-method approaches in capturing the complexity of psychological adaptation.

Taken together, these findings invite a more holistic and integrative conceptualization of resilience—one that incorporates existential, emotional, and relational dimensions. From a practical standpoint, the results suggest that interventions in contexts of collective trauma should target both inner frameworks (such as meaning orientation and optimism) and social pathways (such as fostering belonging and mutual support). Supporting individuals in cultivating meaning-centered engagement—through volunteerism, spiritual expression, and communal routines—may strengthen their capacity to adapt, persevere, and sustain psychological well-being amid prolonged uncertainty.

### 4.1 Limitations and suggestions for future studies

Several limitations of the current study must be acknowledged. First, the cross-sectional nature of this study prohibits interpretation of causality; therefore, we cannot rule out the possibility that individuals with initial higher levels of resilience may less reported fewer suffer from depressive and anxiety symptoms. Accordingly, future research should employ longitudinal designs to investigate it is suggested to further longitudinally investigate the direction of the association found between the reported protective factors in the current study and resilience, to allow better understand they influences resilience ability of the associations we observed between the protective factors and resilience, allowing stronger causal inference. In addition, all focal constructs were assessed by self-report at a single time point, raising the possibility of common-method variance and context-specific response tendencies during the war (e.g., heightened salience of threat cues, socially desirable responding). Some assessments—such as concern about the state’s future—were brief, which limits reliability and content coverage. Future work should incorporate multi-method assessment (behavioral indicators, informant reports, ecological momentary assessment, and, where appropriate, physiological or digital trace data) and employ longer multi-item scales for key appraisals.

Sampling and generalizability are also limited. Recruitment via social media and snowballing in an early war window may over-represent more connected or motivated individuals and under-represent others (e.g., those with restricted access or higher acute burden). Moreover, cultural context (e.g., civic mobilization, communal norms) likely shapes how meaning, optimism, and support are accessed and expressed. Replication in probability samples and across diverse cultural settings and phases of recovery (acute, sub-acute, longer-term) is needed to evaluate stability and boundary conditions.

Future research could also complement our findings with testing interactions (e.g., whether social support buffers high concern) and subgroup moderation (gender, religiosity, exposure) may sharpen interpretation. Furthermore, the qualitative component enriches interpretation but has constraints: brief open-ended responses, potential self-selection into longer narratives, and limits on estimating prevalence from thematic counts. Although inter-rater agreement was high, themes inevitably reflect interpretive choices. Future mixed-methods work should pursue tighter integration (e.g., joint displays, explanatory-sequential designs), link themes prospectively to subsequent quantitative outcomes, and trace how everyday practices identified by participants (volunteering, routines, spiritual practice, physical activity, relationships) evolve alongside trajectories of resilience and distress.

Finally, while the study targeted core protective resources, PTSD—central to war-related mental health—was not measured; future models should incorporate validated PTSD indices, richer exposure metrics, and additional resource/vulnerability markers to allow tests of specificity (e.g., whether optimism and presence of meaning differentially relate to symptom clusters). Finally, although optimism emerged as a significant predictor, its interpretation should be cautious. Optimism can reflect both a relatively stable trait and a state-like response to acute circumstances; distinguishing between these facets—and examining their dynamic interplay with meaning in life and resilience—may yield a more nuanced account of its protective role during ongoing war and crisis.

Overall, the present study offers a unique contribution by examining resilience processes in real time during an ongoing war, rather than retrospectively. In addition, the integration of quantitative and qualitative indicators helped illuminate how psychological resources—such as prioritizing meaning, optimism, and presence of meaning—interact with social support to foster resilience. By highlighting factors that may contribute to resilience during an active conflict, these findings can guide future research and inform practical interventions.

## Data Availability

The raw data supporting the conclusions of this article will be made available by the authors, without undue reservation.
